# Targeting the ACE2 and Apelin Pathways Are Novel Therapies for Heart Failure: Opportunities and Challenges

**DOI:** 10.1155/2012/823193

**Published:** 2012-05-13

**Authors:** Seyyed M. R. Kazemi-Bajestani, Vaibhav B. Patel, Wang Wang, Gavin Y. Oudit

**Affiliations:** ^1^Mazankowski Alberta Heart Institute, University of Alberta, Edmonton, AB, Canada T6G 2S2; ^2^Department of Physiology, University of Alberta, Edmonton, AB, Canada T6G 2H7; ^3^Division of Cardiology, Department of Medicine, University of Alberta, Edmonton, AB, Canada T6G 2G3

## Abstract

Angiotensin-converting enzyme 2 (ACE2)/Ang II/Ang 1–7 and the apelin/APJ are two important peptide systems which exert diverse effects on the cardiovascular system. ACE2 is a key negative regulator of the renin-angiotensin system (RAS) where it metabolizes angiotensin (Ang) II into Ang 1–7, an endogenous antagonist of Ang II. Both the prolonged activation of RAS and the loss of ACE2 can be detrimental as they lead to functional deterioration of the heart and progression of cardiac, renal, and vascular diseases. Recombinant human ACE2 in an animal model of ACE2 knockout mice lowers Ang II. These interactions neutralize the pressor and subpressor pathologic effects of Ang II by producing Ang 1–7 levels *in vivo*, that might be cardiovascular protective. ACE2 hydrolyzes apelin to Ang II and, therefore, is responsible for the degradation of both peptides. Apelin has emerged as a promising peptide biomarker of heart failure. The serum level of apelin in cardiovascular diseases tends to be decreased. Apelin is recognized as an imperative controller of systemic blood pressure and myocardium contractility. Dysregulation of the apelin/APJ system may be involved in the predisposition to cardiovascular diseases, and enhancing apelin action may have important therapeutic effects.

## 1. Introduction

 Angiotensin-converting enzyme 2 (ACE2)/Ang II/Ang 1–7 and apelin/APJ are two important peptide systems with diverse and fundamental cardiovascular protective effects that may prevent or reverse a variety of vascular and cardiac disorders [1–3]. ACE2 is a monocarboxypeptidase which effectively plays a key role as the central negative regulator of the renin-angiotensin system (RAS). ACE2 is of particular interest because it is an essential component of RAS which is possibly implicated in metabolizing angiotensin (Ang) II into Ang 1–7 [4]. These interactions counteract the pathologic effects of Ang II by producing Ang 1–7, that is known to be cardiovascular protective. Ang II impairs cardiovascular function and enhances pressor and subpressor pathologic consequences, and hence Ang 1–7 protects against these pathological processes [1, 2].

**Figure 1:**
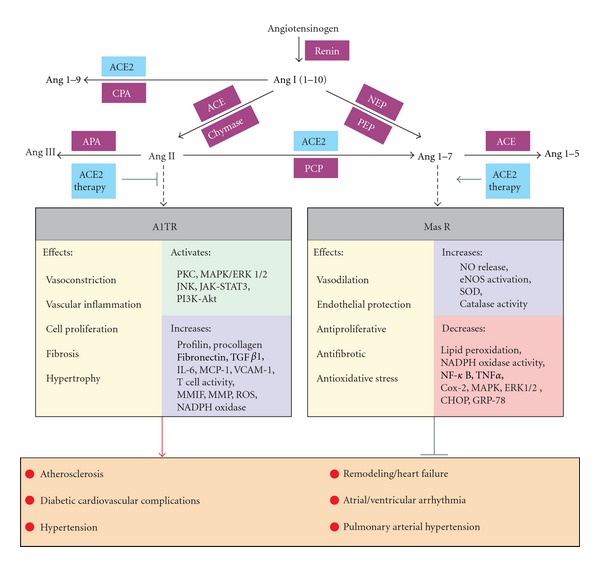
The enzymatic cascade involved in the renin-angiotensin system, key receptor systems, and the biological effects mediated by Ang II and Ang 1–7. Solid black lines, enzymatic pathways; Broken lines, peptide agonist interacting with its key receptor; Green arrow, stimulatory effects; Red arrow, pathologic effects; Green bars; inhibitory effects. ACE2: angiotensin-converting enzyme; Akt: protein kinase B; Ang: angiotensin; APA: aminopeptidase A; AT1R Ang II type 1 receptor; CHOP: CCAAT/enhancer binding protein homologous protein; Cox-2: cyclooxygenase-2; CPA: carboxypeptidase A; eNOS: endothelial synthase; ERK: extracellular signal-regulated kinase; GRP-78: glucose regulated protein; IL-6: interleukin-6; JAK-STAT: Janus Kinase- signal transducer and activator of transcription system; JNK: C-jun-N-terminal kinase; MAPK: mitogen activated protein kinase; Mas R: Ang 1–7 receptor; MCP-1: monocyte cheomattractant protein 1; MMIF: macrophage migration inhibitory factor; MMP: matrix metalloproteinase; NADPH: nicotinamide adenine dinucleotide phosphate; NEP: neutral endopeptidase; NF-kappaB: nuclear factor kappa-light-chain-enhancer of activated B cells; NO: nitric oxide; PCP: prolyl carboxypeptidase (also known as angiotensinase C); PEP: prolyl endopeptidase; PI3K: phosphatidylinositol 3-kinases; PKC: protein kinase C; ROS: reactive oxygen species; SOD: superoxide dismutase; TGF*β*1, transforming growth factor*β*1; TNF *α*: tumor necrosis factor *α*; VCAM-1: vascular cell adhesion molecule-1.

**Figure 2:**
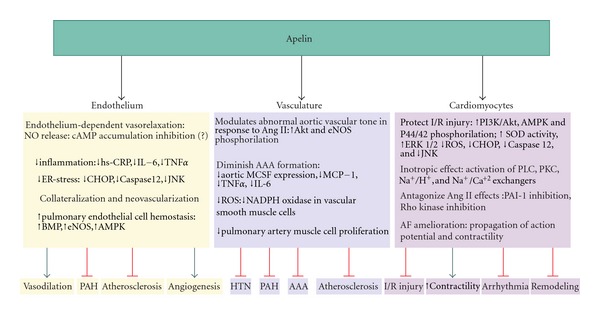
Diverse effects of apelin on cardiovascular system. Black arrows, effects of apelin on various targets; Green arrows and red bars, favorable stimulatory and inhibitory effects of apelin on cardiovascular system respectively; AAA: abdominal aorta aneurysm; AF: atrial fibrillation; Akt: protein kinase B; AMPK: adenosine monophosphate-activated protein kinase; BMP: bone morphogenetic protein; CHOP: CCAAT/enhancer binding protein homologous protein; eNOS: endothelial synthase; hs-CRP: high sensitivity C-reactive protein; ER-stress: endoplasmic reticulum stress; ERK: extracellular signal-regulated kinase; HTN: hypertension; I/R: ischemia reperfusion; IL-6: interleukin 6; JNK: C-jun-N-terminal kinase; MCSF: macrophage colony stimulating factor; MCP-1: monocyte cheomattractant protein 1; NADPH: nicotinamide adenine dinucleotide phosphate; NO: nitric oxide; PAH: pulmonary arterial hypertension; PI3K: phosphatidylinositol 3-kinases; PKC:protein kinase C; PLC: Phospholipase-C; ROS: reactive oxygen species; SOD: superoxide dismutase; TNF*α*: tumor necrosis factor.

Apelin is an endogenous peptide that is widely expressed in various organs as a 77 amino acid preproapelin. Several active fragments of apelin have been known (apelin-36, apelin-19, apelin-17, apelin-16, apelin-13, and apelin-12) which relatively share similar biological activities. In addition to the possibility of application of apelin as a heart failure (HF) biomarker, apelin also has direct biological effects including vasodilatory and inotropic effects [5]. Several previous studies have shown the cardioprotective effect of ACE2 and apelin in all three steps of primary, secondary and tertiary prevention of HF (Figures 1 and 2). In this paper we summarize the current literature regarding the cardiovascular effects of ACE2 and apelin and their possible therapeutic applications.

## 2. Role of ACE2 and Apelin in Systemic and Pulmonary Hypertension

 Several previous studies demonstrated that ACE2 can modulate blood pressure. Daily infusion of recombinant human ACE2 (rhACE2) (2 mg/kg^−1^/d^−1^) reduces the Ang II-induced hypertension in mice, by reducing Ang II-mediated activation of enhanced extracellular signal-regulated kinase 1/2 (ERK1/2), protein kinase C (PKC) pathways and renal fibrosis [6]. It is suggested that ACE2 expression is decreased in the spontaneously hypertensive rats before the marked onset of hypertension [7]. It is believed that impaired renal level of ACE2 contributes to hypertension in humans [8]. Exposure of cultured human umbilical artery smooth muscle cells (HUASMCs) to Ang II results in a significant increase in the mRNA and protein expression of profilin-1, recently linked to cytoskeleton remodeling by activation of the hypertrophic signaling through mitogen activated protein kinase (MAPK), ERK1/2 and C-jun-N-terminal kinase (JNK), in conjunction with reduced ACE2 activity [9]. Enhanced profilin-1 expression and MAPK signaling in HUASMCs in response to Ang II was noticeably reduced by rhACE2 in an Ang 1–7-dependent manner [9]. Improvement of ACE2 expression and reduced profilin-1 levels were associated with an overt suppression of ERK1/2 and JNK phosphorylation in aortas of spontaneously hypertensive rats [9].

 ACE2 overexpression results in increased expression of various antihypertensive components of the RAS including Ang 1–7/Mas and AT2R. Vascular transgenic overexpression of ACE2 results in reduction of arterial blood pressure [10, 11] and an attenuated response to Ang II infusion [10]. Central nervous system overexpression of ACE2 also was proved to be protective against Ang II induced hypertension [12]. Wysocki et al. (2010) showed that acute Ang II infusion-induced hypertension can be prevented by rhACE2 pretreatment (2 hrs before the Ang II infusion) in mice. This antihypertensive effect of rhACE2 was associated with a decrease in plasma Ang II and increases in plasma Ang 1–7 levels. However, the antihypertensive effect of rhACE2 was shown to be independent of Ang 1–7 in this study [13]. Wysocki et al. concluded that circulatory decrease of Ang II was the driving cause of decreased hypertension, rather than increased levels of Ang 1–7 [13]. This acute antihypertensive effect of ACE2 seems promising for management of the patients with hypertension.

 Reduced circulating levels of apelin have been demonstrated in the patients with essential hypertension [14, 15]. Genetic variation in apelin likely contributes to essential hypertension and the onset of aged hypertension [16]. Tatemoto et al. (2001) showed that arterial pressure after the administration of apelin-12, apelin-13, and apelin-36 at a dose of 10 nmol/kg resulted in a reduction in arterial blood pressure [17]. Cheng et al. (2003) examined dose response curves of apelin (10, 20, and 40 nmol/kg) in rats and concluded that apelin is an arterial and venous dilator *in vivo* [18]. Japp et al. (2008) showed nitric oxide (NO)-dependent vasodilatory effect of apelin in 24 healthy volunteers *in vivo* [19]. However, the long-term effects of manipulating the apelin pathway and its effect on blood pressure are unknown.

 Several studies focused on the role of ACE2 and apelin in amelioration of pulmonary arterial hypertension (PAH). Ang II is of fundamental importance to development and progression of PAH, due to its vasoconstrictive, fibrotic, and proliferative effects [20]. Left ventricular failure/remodeling is one of the frequent consequences of PAH which may aggravate the global function of the heart. The ability of ACE2 to combat the fibrosis/proliferative effects of Ang II on lung and right ventricle (RV) supported the beneficial role of ACE2 in PAH treatment [20]. ACE2 overexpression [21] and ACE2 activation by 1-[(2-dimethylamino) ethylamino]-4-(hydroxymethyl)-7-[(4-methylphenyl) sulfonyl oxy]-9H-xanthene-9-one (XNT) [22] ameliorate RV systolic failure, decrease the adverse effects of Ang II, and attenuate pro-inflammatory cytokines in PAH mice. There is promising evidence of rhACE2 on improvement of RV load-stress (pulmonary artery banded (PAB)) of early HF [23]. rhACE2 administered to PAB mice for two weeks prevented load-induced RV systolic and diastolic dysfunction [23]. The exceptional increasing effect of rhACE2 on RV [23] shows the remarkable property of ACE2 for potentially complex patients with isolated RVHF.

 Apelin/APJ is highly expressed in pulmonary vasculature [24]. Chandra et al. (2011) reported significantly lower serum apelin levels in patients with PAH compared to control subjects [25]. Apelin expression also decreases in the pulmonary endothelial cells of the patients with PAH [26]. It was shown that PAH in mice may originate from the disruption of apelin signaling which is mediated by decreased activation of adenosine monophosphate-activated protein kinase and endothelial synthase (eNOS) [25]. Pyr-Apelin-13 treatment has been reported to downregulate Ang II and endothelin-1 and could therefore attenuate RV hypertrophy and diastolic dysfunction in rats with PAH [27]. Alastalo et al. (2011) have elegantly shown that apelin could have both autocrine and paracrine effects against PAH in pulmonary vasculature [26]. Bone morphogenetic protein-mediated apelin autocrine production results in enhanced pulmonary arterial endothelial survival, proliferation, and migration which can protect the vasculature against PAH. Importantly, apelin autocrine function against PAH is based on attenuation of the pulmonary arterial smooth muscle cells response to growth factors and by promoting apoptosis [26].

## 3. Role of ACE2 and Apelin in Diabetes (DM) and Diabetic Cardiovascular Complications

 Attenuation of Ang II-induced glomerular mesangial cell proliferation, oxidative stress, and collagen IV protein synthesis is thought to be the critical steps in ACE2-related protection of diabetic nephropathy [28, 29]. Even in non-diabetic mice loss of ACE2 contributes to increase in renal lipid peroxidation product formation and activation of MAPK and ERK 1/2 in glomeruli [30]. ACE2 mRNA has been shown to be reduced by more than half in both the glomeruli and proximal tubules of the diabetic patients compared to controls [31]. ACE2 treatment is believed to be critical in protection against diabetes induced nephropathy. rhACE2 treatment attenuated high glucose in cultured primary rat mesangial cells [32]. The potential role of Ang II in the induction of kidney injury in ACE2 knocked out mice has been reported [33]. In addition to the possibility of renal protective role of rhACE2 due to its antihypertensive effect in diabetic subjects, rhACE2 also attenuates diabetic nephropathy via blockade of Ang II-induced nicotinamide adenine dinucleotide phosphate (NADPH) oxidase activity in mesangial cells [32]. Bindom et al. (2010) have elegantly shown that ACE2 gene therapy in mice results in reduced fasting blood glucose and glucose tolerance improvement in a diabetic mice model [34]. They also proved that ACE2 overexpression in diabetic mice significantly reduces apoptosis which can be prevented by Ang 1–7 receptor blockade [34], further supporting the link between ACE2 and diabetic glycemic control [34, 35].

 Several studies have shown an association between apelin levels and overt diabetes [36]. Erdem et al. (2008) demonstrated that plasma apelin was lower in newly diagnosed and untreated patients with DM II compared to healthy controls [37]. Soriguer et al. (2009) showed the association between apelin levels and glucose concentrations and insulin sensitivity in diabetic patients suggesting the role of apelin in diabetes pathogenesis [38]. Furthermore, diabetic mice exhibited downregulation of apelin receptors and depressed aortic vascular tone [39]. However, Rittig et al. (2011) examined the association between apelin and atherosclerosis indicators (intima media thickness) in 344 subjects with an increased risk for DM II and did not show any association to diabetes risk pattern [40].

 The effect of apelin in DM control has been shown in some animal studies. Intracerebroventricular injection of apelin in mice leads to improved glucose homeostasis via NO-dependent pathway [41]. Injection of apelin-13 (400 pmol/kg) for 10 weeks considerably reduced the pancreas endoplasmic reticulum (ER) stress in Akita mice, a model of DM I, which leads to modification of pancreatic islet mass reduction and preservation of insulin content [42]. This important effect of apelin-13 in type 1 diabetes was mediated by inhibition of inositol requiring enzyme 1-*α* and JNK pathways, suggesting apelin effects on two important pathways of ER stress and cell death, respectively [42]. In mice with metabolic syndrome, apelin restores glucose tolerance and increases glucose utilization [43]. Apelin treatment has a favorable effect on vascular function in diabetic mice. Apelin treatment remarkably adjusts the abnormal aortic vascular reactivity in response to Ang II and acetylcholine in DM II mice by increasing the phosphorylation of Akt and eNOS [39]. Apelin may improve the glycemic status and insulin sensitivity of the patients and also can ameliorate vascular functions of diabetic patients.

## 4. Role of ACE2 and Apelin in Vascular Inflammation and Atherosclerosis

 The effect of vascular inflammation in the initiation and progression of atherosclerosis/cardiovascular diseases has been well recognized. Gene transfer of ACE2 suppresses the expression of macrophage migration inhibitory factor, a proinflammatory cytokine associated with insulin resistance, in endothelial cells stimulated by Ang II [44]. ACE2 deficiency contributes to enhanced atherosclerotic plaque formation and also increases proinflammatory cytokines, including interleukin-6 (IL-6), monocyte chemoattractant protein-1 (MCP-1), and vascular cell adhesion molecule-1, which defined the key role of ACE2 in modulation of inflammation-dependent atherosclerosis development [44]. Remarkable effects of rhACE2 on attenuation of Ang II-induced T-lymphocyte-mediated inflammation can be considered as an evidence for antiinflammatory/anti-atherosclerotic effects of ACE2 [45].

 ACE2 reduced atherosclerosis progression in apolipoprotein E knockout mice probably by inhibition of reactive oxygen species (ROS) subsequent activities [46]. Association between ACE2 antiatherosclerotic effects and disturbance of Ang II/Ang 1–7 peptides has been proved [47]. Zhang et al. (2010) suggested the downregulation of the ERK-p38, Janus kinase-signal transducer and activator of transcription system (JAK-STAT), and Ang II-induced ROS-nuclear factor kappa light chain enhancer of activated B cells (NF-kappaB) signaling pathways and upregulation of the phosphatidylinositol 3 kinases-protein kinase B (PI3K-Akt) pathway subsequent to ACE2 therapy in rabbits [48]. It has been shown that overexpression of ACE2 can readily stabilize the atherosclerotic plaques probably due to protective effects of Ang 1–7 [49]. Hence we concluded that ACE2 is essential to prevent or delay the development of atherosclerosis. The plaque stabilizing effect of ACE2 seems promising for prevention of acute coronary syndromes (ACS).

 Plasma levels of apelin inversely correlated to inflammatory markers (C-reactive protein and IL-6) in hemodialysis patients [50]. Apelin treatment in mice models of abdominal aorta aneurysm (AAA) clearly demonstrated its anti-inflammatory effects that could attenuate AAA formation [51]. Injected apelin can reduce the mRNA levels of pro-inflammatory markers (MCP-1, macrophage inflammatory protein-1*α*, IL6 and tumor necrosis factor-*α*) [51]. Apelin attenuates ultraviolet B-induced edema and inflammation in mice and play an important role in stabilization of the tissue [52]. Apelin exerts acute anti-inflammatory effects on the vascular system; the results are promising and if these results can be extrapolated in chronic models, it can be a proper therapeutic modality for prevention of inflammation in the process of atherosclerosis. Pitkin et al. (2010) have shown an increase in apelin expression in atherosclerotic coronary artery, with the additional peptide localizing to the atherosclerotic plaque [53]. Apelin receptor was also found to be present within the atherosclerotic plaque and to have a similar distribution to its ligand [53]. Increased content of apelin and its receptor might be an indicator of increased anti-inflammatory activation of macrophages thereby limiting plaque instability. Due to lack of data and contradictory findings, the exact role of apelin on atherosclerosis plaque remains inconclusive.

## 5. Role of ACE2 and Apelin in Angiogenesis

 The association between ACE2 and cardiac angiogenesis during the HF process has not been studied. A few studies in the cancer field have supported an important role of ACE2 in angiogenesis [54, 55]. The probable adverse antiangiogic effects of ACE2 in HF remain unclear. Apelin angiogenenic effects have been proved in few animal studies; however none of the studies targeted the effect of apelin on heart angiogenesis. Tiani et al. (2009) showed the remarkable effect of apelin on portosystemic collateralization and splanchnic neovascularization in portal hypertensive rats [56]. Treatment of human umbilical vein endothelial cells with apelin dose-dependently augments angiogenic responses [57]. Kidoya et al. (2010) indicated that apelin together with vascular endothelial growth factor (VEGF) efficiently induced functional vessels larger than with VEGF alone, in the hind limb ischemia model of mice [58]. Apelin is required factor for hypoxia-induced retinal angiogenesis in mice [59]. Available data imply that apelin is an effective factor in angiogenesis; however none of the studies have targeted coronary vessels to test the effect of apelin on their angiogenesis. If the effect of apelin on cardiac collateralization is proved in future studies, it can be considered as a valuable factor for the patients with HF, in particular ischemic HF. The probable off-or on-target effects of apelin on other organs angiogenesis, for example, retinal neovascularization/angiogenesis make interpretation problematic.

## 6. Role of ACE2 and Apelin in Post-Myocardial Infarction (MI) Remodeling

 Post-MI remodeling and coronary artery disease are now the most common cause of HF [60]. In ACE2 deficient mice, MI leads to enhanced activation of the RAS resulting in increased cardiac mortality, adverse ventricular remodeling, and aggravated systolic function [61]. ACE2 contributes to generation of myocardial Ang 1–7 after MI which may protect the heart from ischemic consequences [61]. Loss of ACE2 in post-MI mice is associated with increased Ang II levels and ROS production. This is followed by increased matrix metalloproteinase (MMP) activation, inflammation, and activation of MAPK in ACE2-deficient hearts [61]. Der Sarkissian et al. (2008) analyzed the rats that received an intracardiac injection of lentivirus containing ACE2 cDNA followed by coronary artery ligation and found the ischemic protection of myocardium by ACE2 24 hours after the ischemic event compared to the control mice [62]. The effect of ACE2 overexpression on attenuation of left ventricular fibrosis/remodeling and systolic function was also shown one month after MI [63].

 Ang II antagonist infusion for 28 days after MI results in augmented ACE2 cardiac mRNA in normotensive rats, which may be related to direct blockade of AT1R or the probable contribution of increased Ang 1–7 [64]. Attenuated cardiac hypertrophy and improved ventricular contractility both were chronic antiremodeling effects of ACE2 on ACS-induced ischemia [64]. Treatment of the rats for a same period (28 days) with C16, a selective non-peptidic ACE2 inhibitor, at a dose that inhibited myocardial ACE2 activity was associated with a significant increase in MI infarct size [65]. It seems that acute and long-term effects of ACE2 limits the infract size following ACS. ACE2 is capable to produce Ang 1–9 from Ang I [4]. Ocaranza et al. (2010) proved the efficacy of Ang 1–9 in attenuation of post-MI remodeling [66]. Further studies are needed to evaluate the effect of ACE2 on Ang 1–9 increase and its remodeling-attenuation in coronary artery disease.

 Decreased levels of apelin-36 during 5 days interval following ST elevation MI have been reported [67, 68]. Weir et al. (2009) also confirmed depressed level of apelin early after MI. They showed significant increase of apelin from base line to 24 weeks after MI [69]. None of these studies found any relation between apelin levels and left ventricular function [67–69]. Kadoglou et al. (2010) showed that both groups of patients with unstable angina and acute MI had significantly lower level of apelin compared to the patients with asymptomatic coronary artery disease [70]. According to our knowledge, only decreased levels of apelin after ACS have been proved without any relation with structural dysfunction of the heart. The therapeutic effect of apelin in the management of patients with ACS has not been examined. Evaluation of apelin role before and after acute events and its role in plaque stabilization seems to be complicated in animals, as there is no model of unstable atherosclerotic plaque-induced ACS.

 The precise role of ACE2 in protection of the heart and kidney against ischemia-reperfusion (I/R) injury has not been elucidated. However, according to the association between ACE2 and MAPK [61], ACE2 protective effects against I/R injury seem probable. Simpkin et al. (2007) for the first time demonstrated the protective effects of apelin against I/R injury in rodents through the reperfusion injury salvage kinase (RISK) pathway activation [71]. In murine Langendorff model of I/R injury, apelin-13 could increase Akt and ERK1/2 phosphorylation as well as increase Akt activity at 5 and 10 min of reperfusion [72]. Activation of Akt and ERK1/2, two important members of RISK pathway, can potentially protect the heart against I/R injury [72]. Administration of apelin can partly block the ER stress-dependent apoptosis activation in rat models of I/R injury at 2 h of reperfusion which results in the heart protection against I/R injury [72]. This protection against I/R injury remained significant during time-related changes at 24 h of reperfusion [72]. Administration of apelin (30 pM) in Langendorff model of perfused isolated rat hearts favorably preserves the impaired cardiac function [73]. In rat cultured cardiomyocytes, the antioxidant activity of apelin is thought to be largely due to inhibition of ROS production, malonaldehyde activity and lactate dehydrogenase leakage and also activation of superoxide dismutase [73]. According to several previous investigations, apelin can considerably protect the heart against I/R injury. However, apelin effects against I/R injury have not been tested in humans. This promising effect of apelin can be applied in humans in the conditions that I/R injury can impair the heart function and might be a leading cause of HF. Application of apelin during the percutaneous coronary intervention and coronary artery bypass graft immediately and in early days after the procedure may have therapeutic benefits.

## 7. Role of ACE2 and Apelin in Arrhythmias

 Atrial fibrillation (AF) is one of the most common arrhythmias among the patients with cardiovascular diseases. Downregulation of both the mRNA and protein level of ACE2 has been shown to be associated with the development of AF in a rapid pacing-induced model [74]. They also showed higher expression of ERK1/2 cascade and also increased expression of collagen I protein in the atrial tissues with AF that is related to increased local Ang II [74]. It could be concluded that ACE2 treatment can easily protect the heart against the AF complications including fibrosis and remodeling. However the results of Donoghue et al. paper (2003) seem contradictory [75]. Donoghue et al. (2003) reported that ACE2 overexpression in the heart leads to probable gap junction remodeling, resulting in severe electrophysiological disturbances (sustained ventricular tachycardia and terminal ventricular fibrillation) and high incidence of sudden death in transgenic mice [75]. The results of Donoghue et al. has not been confirmed again by other studies, and several investigations have used ACE2 transgenic mice without increased sudden cardiac death.

 Serum apelin levels are lower in the patients with AF compared to controls [76, 77]. Falcone et al. (2010) measured apelin in 93 patients with persistent AF before successful external electrical conversion. Patients with apelin levels below the median showed a hazard ratio of 3.1 of arrhythmia recurrence compared to those with high apelin levels [78]. Low plasma apelin is an independent prognostic factor for arrhythmia recurrence in the patients with AF under antiarrhythmia medication [78]. Apelin, due to its effect on the propagation of action potential and contractility in cardiomyocytes, is thought to modulate the pathophysiology of AF [79]. Apelin increases sarcomere shortening in normal as well as failing cardiomyocytes [79]. Moreover, apelin augments conduction velocity in monolayers of cultured neonatal rat cardiac myocytes [79]. According to our knowledge, the level of apelin has been investigated only in the patients with AF, and other forms of arrhythmia have not been investigated.

## 8. Role of ACE2 and Apelin in HF: Therapeutic Potentials

 The relationship between ACE2 deficiency and failure of heart function including pathological hypertrophy, ventricular remodeling, and systolic dysfunction is well described [6, 80, 81]. In ACE2 deficient mice model of pressure overload, increased Ang II leads to severe cardiac hypertrophy [80, 82], increased activity of MAPK [82], activation of the NADPH oxidase system and oxidative stress-induced augmented MMP-mediated degradation of the extracellular matrix [80]. rhACE2 has antifibrosis properties and may attenuate expression of Ang II-induced procollagen, transforming growth factor-*β*1, and fibronectin [6]. The attenuating effect of rhACE2 on systolic and diastolic dysfunction [6, 81] is thought to be largely due to Ang II inhibition [6]. rhACE2 treatment blocks the Ang II-mediated activation of PKC-*α*, PKC-*β*1, ERK 1/2 and JAK-STAT in wild-type mice [6]. Ferreira et al. (2011) showed that chronic XNT infusion, an ACE2 activator, was associated with decreased cardiac collagen content, increased cardiac Ang 1–7 immunostaining and a reduction in ERK phosphorylation [83]. Ang 1–9, a known product of ACE2 activity in RAS, results in considerable reduced hypertension-induced cardiac fibrosis through modulation of collagen I expression [84]. Furthermore, Ang 1–9 attenuates Ang II-inducfed cardiomyocyte hypertrophy [85]. ACE2 by inhibiting several remodeling pathways can attenuate Ang II and pressure-overload-induced cardiomyopathy.

 In HF there is hyperactivity of the sympathetic nervous system which chronically may have adverse effect on cardiac function. ACE2, Ang 1–7, and the Mas receptor exist in the brain; however, controversy remains over their relation with the cardiovascular functions [86]. There is growing interest in the application of ACE2 in central nervous system. Xiao et al. (2011) showed that central ACE2 overexpression applies a considerable cardiac protective effect in the mice model of HF which was associated with a significant decrease in sympathetic biomarkers [87]. Brain selective human ACE2 over expression also showed to be effective for management of hypertension in transgenic mice [88]. Direct effects of central ACE2 treatment on HF improvement and also its role in hypertension attenuation are both acceptable evidence for the efficacy of ACE2 on HF management.

 Arrhythmia also may occur in the patients with HF and can worsen the structural and functional status of the heart in these patients. The protective effect of ACE2 against arrhythmia in the failed heart is a subject of debate. The importance of the balance between ACE-Ang II-AT1R axis and the ACE2-Ang 1–7-Mas receptor axis in the regulation of heart cell volume has been suggested [89]. The key role of Ang 1–7 in decreasing the cell volume which results in decline in activation of swelling-activated chloride current (*I*_Clswell_) suggesting a likely contribution of ACE2 in prevention of major post-ischemic cardiac arrhythmias [89]. However, De Mello (2009) mentioned the probable effect of overexpressed ACE2 on generation of early afterdepolarization especially in the patients with HF [90]. Regarding the probable arrhythmogenic effects of ACE2 in the patients with HF, it seems that precise dose adjustment based on the severity of HF may prevent this side effect.

 There is growing interest regarding the protective role of apelin in HF development. Apelin level is considerably reduced in the patients with HF [91–93]. Several studies showed high expression of apelin/APJ in the heart and in vascular systems of rodents and humans [24, 93, 94]. The mechanisms by apelin reduction causes HF are becoming clearer. Gao et al. (2009) reported significant increase of apelin as an indicator of improved cardiac function from 3 to 21 days after bone marrow mononuclear cell transplantation in the patients with HF through autocrine and paracrine mechanisms [92]. Apelin reduces left ventricular preload and afterload in rodents [95] and is known to be a strong positive inotropic agent [53, 94, 96] that could be outstandingly helpful in treatment of HF. Apelin mutant mice develop HF associated with aging and pressure overload [97]. Infusion of apelin-13 [98] and apelin-12 [99] enhances myocardial function of the left anterior descending artery ligation model of HF in rats. Perfusion of isolated rat hearts with apelin-16 caused an inotropic effect with a similar time course to endothelin-1 [94]. Interestingly, apelin can present a gradually developing but sustained inotropic effect [94], which is a significant difference compared to classical *β*-adrenergic effects. Apelin administration to the rats in ischemic HF significantly attenuates diastolic dysfunction [96]. The involvement of phospholipase-C, PKC, Na^+^/H^+^ and Na^+^/Ca^2+^ pumps has been proved in positive inotropic effects of apelin [94, 100, 101]. The interaction between apelin and two important regulatory pumps has been proposed to be contributed to increased inotropic effects of apelin by restoration of calcium in the cardiomyocyte cytosol.

 Japp et al. (2010) investigated the acute cardiovascular effect of intrabrachial infusion of (Pyr (1)) apelin-13 in the patients with chronic HF and healthy volunteers and found vasodilatation in patients and control subjects [102]. Systemic infusions of (Pyr(1)) apelin-13 (30 to 300 nmol/min) result in elevated cardiac index, lowered mean arterial pressure and peripheral vascular resistance in HF patients and healthy control subjects [102]. Intracoronary bolus of apelin-36 leads to increased coronary blood flow and reduced peak and end-diastolic left ventricular pressures [102]. These remarkable peripheral and coronary vasodilatation effects of apelin and also its effect on cardiac output increase shows apelin as a novel medication for the patients with HF. Decreased density of apelin receptors in the heart tissues with cardiomyopathy may block the inotropic effect of apelin on the heart [53]. APJ gene also has been suggested as a modifier gene for idiopathic dilated cardiomyopathy [103]. Further investigations should focus on combination of apelin therapy with apelin receptor agonists. Synergetic effects of apelin with APJ agonists may increase the efficacy of apelin therapy for the patients with HF. However, these findings are not consistent with Atluri et al. (2007) findings in rats [98]. Atluri et al. (2007) showed increased APJ protein levels in myocardium of the rats with HF [98]. Pitkin et al. (2010) suggested [Glp65,Nle75,Tyr77][125I]-apelin-13 as a potent agent with high-affinity, saturable and reversible affect which might reflect its therapeutic efficacy in future [53].

 The difference between APJ in myocardium and arterial system of the HF patients is not completely defined. Due to the dual ionotropic and hypotensive effects of apelin on the patients with HF, we need to be cautious about the application of this agent in the clinical setting. APJ agonist drugs may increase the efficacy of apelin therapy in the HF patients; however it is not clear whether APJ agonist drugs will increase the arterial hypotensive effects of apelin. Experimental evidence has established an association between Ang II and development of HF [45, 81]. Chun et al. (2008) proved that apelin signaling can antagonize Ang II actions in vascular disease by NO production and inhibiting Ang II cellular signaling [104]. Generally, apelin modulates Ang II-induced cardiovascular fibrosis [105] which may be linked to apelin ability to inhibit the plasminogen activator inhibitor type-1 production resulting in secondary changes in the expression of matrix proteins and degrading enzymes [105]. Apelin-13 inhibits Ang II-induced vascular contraction mainly through NO-dependent pathways [106]. There is evidence that apelin can be increased by blocking RAS [107] which may contribute to AT1 blocker treatment in the clinical setting [108]. This evidence led to the notion that apelin therapy may ameliorate RAS-related HF aggravating effects.
